# Isolation of *Escherichia coli* with a combination of carbapenemases (*bla*_NDM_ *+bla*_OXA-48-like_ ) in a hospital in Huancavelica, Peru

**DOI:** 10.17843/rpmesp.2025.423.14193

**Published:** 2025-08-25

**Authors:** Dany Victorio-López, Wendi Pompilio, Jhonatan Carrera-Donayre, Juan Ramírez-Illescas, Raquel Maraza-Huarachi, Maritza Mayta-Barrios, Diana Flores-León, Martin Yagui, Luis Pampa-Espinoza

**Affiliations:** 1 Hospital Departamental de Huancavelica, Ministerio de Salud, Huancavelica, Peru. Hospital Departamental de Huancavelica Ministerio de Salud Huancavelica Peru; 2 Laboratorio de Bacteriología Clínica, Instituto Nacional de Salud, Lima, Peru. Laboratorio de Bacteriología Clínica Instituto Nacional de Salud Lima Peru; 3 Unidad de Intervenciones Estratégicas, Instituto Nacional de Salud, Lima, Peru. Unidad de Intervenciones Estratégicas Instituto Nacional de Salud Lima Peru

Mr. Editor. Infections caused by multidrug-resistant bacteria constitute a global public health problem. In 2019, before the start of the COVID-19 pandemic, 4.5 million deaths attributable to resistant infections were described ^1^. The indiscriminate use of antibiotics, coupled with insufficient control measures for their prescription, has created the perfect scenario for the progressive increase of multidrug-resistant infections in Peru ^2^. The COVID-19 pandemic exacerbated this problem, with it being observed that more than 50% of hospitalized patients received antibiotics without a clear prescription ^3^. Faced with this situation, it is imperative to strengthen strategies such as antimicrobial stewardship programs (ASPs), which have been reported to be operational in only 28.8% of health facilities in Peru as of 2022 ^4^. Likewise, it is necessary to strengthen diagnostic capacities by implementing laboratories with adequate infrastructure, advanced technology, and specialized microbiological tests, which allow for a rapid and precise diagnosis of multidrug-resistant germs in Peruvian hospitals. A timely diagnosis, along with appropriate treatment, significantly increases the chances of survival in adult patients with complicated infections ^5^. In this context, we present the following finding as an example of the microbiological analysis performed on a patient hospitalized in a public health facility in Peru.

The finding pertains to a 53-year-old male patient, referred from a health facility in the district of Yauli in Huancavelica, due to an episode of respiratory decompensation, for which he was admitted to the hospital’s intensive care unit (ICU) for specialized evaluation, management, and monitoring. During his stay, a bronchial secretion sample was obtained for microbiological culture. After three days, *Escherichia coli* was isolated with a multidrug-resistance profile to penicillins, cephalosporins, aminoglycosides, fluoroquinolones, and carbapenems. Antimicrobial susceptibility was determined by an automated system (VITEK®). Given the relevance of the finding, the hospital’s laboratory team sent the bacterial strain for study and confirmation to the Clinical Bacteriology Laboratory of the National Institute of Health (INS) of Peru, where the strain was confirmed by the MALDI-TOF system and resistance mechanisms by disk diffusion synergy tests. Finally, the conventional multiplex PCR system detected the combination of the resistance genes *bla*_NDM_ + *bla*_OXA-48-like_.

Carbapenem-resistant species are considered critical priority pathogens according to the list of priority pathogens by the World Health Organization ^1^. If the upward trend in resistance rates among gram-negative bacilli continues, this situation could lead to an endemic scenario, with mortality rates reaching up to 50% ^6^. According to Mayta-Barrios *et al.*, an increase in reports of germs with carbapenem resistance has been documented in Peru before the COVID-19 pandemic; furthermore, there is evidence of a growing dissemination and geographical expansion of these cases. The reported risk factor was using antibiotics before hospital admission ^6^. Regarding the microbiological analysis, the recommendations of the epidemiological alert AE-001-2022 from the National Center for Epidemiology, Prevention, and Disease Control were followed ^7^. First, hospital-level studies were conducted through screening for the detection of carbapenemases. This was carried out based on the report from the bronchial secretion culture, in which a carbapenem-resistant *E. coli* strain was identified in the antibiogram. Second, a disk synergy test was performed, which detected a metallo-β-lactamase type carbapenemase. Third, the presence of resistance genes was confirmed by polymerase chain reaction (PCR), determining the existence of *bla*_NDM_ + *bla*_OXA-48-like_ type genes according to the analysis performed at the INS (Table 1).

**Table 1:** Proposed and implemented actions in laboratories, hospitals, LARESA, DIRESA/DIRIS, and the National Institute of Health (INS) for studies of critical priority bacteria.

Activities	Hospital Laboratory	Regional Public Health Laboratory and DIRESA/DIRIS	Clinical Bacteriology Laboratory of the INS
Proposed actions for each jurisdiction	Perform manual and/or automated phenotypic study (culture and antibiogram). If resistance to carbapenems exists (CLSI), coordinate sending the strain to LARESA. Implement rapid methods such as Blue-Carba, direct Carba NP, disk synergy (EDTA/APB/CZA), lateral flow immunoassay, etc.	Ensure rapid transport and good preservation of shipment to INS (quality control). Manage and assist the hospital in managing the annual budget with the regional government for resistant germ laboratory supplies. Coordinate with the epidemiology unit of the HCF and DIRESA/DIRIS to report the regional HAI outbreak to generate a hospital epidemiological alert. Provide early technical assistance in the face of HAI outbreaks in hospitals within its jurisdiction.	Perform confirmatory molecular studies (PCR) and species confirmation (MALDI-TOF) of submitted strains. Train microbiology teams from hospitals and the region in rapid methods. Report and coordinate results with the region and hospital through NetLab.
Actions taken in the report.	Isolation of an automated culture of *E. coli* pan-resistant to tested antibiotics such as penicillins, cephalosporins, and carbapenems, etc. (supplementary material 1).	Reception of the sample from the hospital and transport to the INS of Peru.	Susceptibility testing of *E. coli* and disk synergy (supplementary material 2). Confirmatory molecular PCR test for the detection of *E. coli* carbapenemases _NDM_ + *bla*_OXA-48-like_ (supplementary material 3).

The table describes the actions taken in the finding of the *Escherichia coli* strain with resistance to carbapenems and dual carbapenemase production. Source: CDC Peru (7).

LARESA: Regional Public Health Laboratory, DIRESA: Regional Directorate of Public Health, DIRIS: Directorate of Integrated Health Networks of Lima, GORE: Regional Government, EESS: health care facility, INS: National Institute of Health, PCR: polymerase chain reaction, IAAS: healthcare-associated infections.

The combination of carbapenemases has begun to be reported in Latin America following the COVID-19 pandemic, which has led to the issuance of epidemiological alerts in the region. Due to its high epidemiological risk of generating intra-hospital outbreaks and its association with high mortality rates in infected patients, this situation demands the reinforcement of diagnostic efforts at the hospital level, with confirmation at the regional or national level ^8^. In Peru, findings of carbapenemase combinations have begun to be reported ^9^. This enzymatic combination represents an additional risk due to the presence of plasmids in its genomic structure, which facilitate the horizontal transfer of resistance genes between different bacterial species. For this reason, infection prevention and control measures must be reinforced, including hand hygiene for healthcare personnel, contact precautions, and standard precautions, as well as optimizing the cleaning and disinfection of hospital environments.

For the analysis of this strain, there was communication between the hospital laboratory and the INS Clinical Bacteriology Laboratory with feedback on the confirmatory analyses. Hospitals with their own budget and support from the Regional Government (GORE) must have a microbiology laboratory equipped with manual and automated methods and rapid tests, such as Blue-Carba, direct Carba NP, disk synergy (EDTA/APB/CZA), lateral flow immunochromatographic assays, among other rapid diagnostic technologies. The availability of these tests would allow for faster decision-making for the critically ill hospitalized patient, which could reduce hospital stay and improve survival. In this report, limitations in clinical information were identified due to the retrospective nature of the analysis and delays in the referral of samples.

In conclusion, hospitals, regional authorities, and the national level require a real, sustained, and coordinated commitment, which implies constant work from laboratories when critical priority germs are suspected. It is recommended that microbiology teams manage the timely submission of priority strains to the reference laboratory for their genotypic characterization. This effort will allow for a better understanding of the current landscape of hospital-acquired infections caused by multidrug-resistant bacteria in Peru, and will facilitate more timely, evidence-based clinical and public health decision-making.
