# Supplementary material for: Isolation of Escherichia coli with a combination of carbapenemases (bla NDM +bla OXA-48-like ) in a hospital in Huancavelica, Peru
Source: Rev Peru Med Exp Salud Publica. 2025 Aug 25;42(3):333–5. doi: 10.17843/rpmesp.2025.423.14193 (PMC12679978; doi:10.17843/rpmesp.2025.423.14193)
Supplement: Supplementary material. — Available in the electronic version of the RPMESP. [file rpmesp-42-03-14193-s001.docx]

**Materia suplementaria 1:** Reporte de cultivo automatizado de hospital.

_______________________________________________________________________

**Resumen de Cultivo**:

Muestra: Secreción bronquial

Organismo: *Escherichia Coli*

Probabilidad: 96% (Vitek 2)

**Antibiótico: MIC / Interpretación**

Amp/Sulb: > 32 / R

Pipetazo: > 128 / R

Cefazolina: > 64 / R

Ceftriaxona: > 64 / R

Meropenem: > 16 / R

Imipenem: > 16 / R

Ertapenem: > 8 / R

Amikacina: > 64 / R

Ciprofloxacino: > 4 / R

_______________________________________________________________________

**Fuente**: Área de microbiología del Hospital Departamental de Huancavelica

**Materia suplementaria 2**: Prueba de susceptibilidad a Escherichia coli y mecanismos fenotípicos de resistencia (INS).


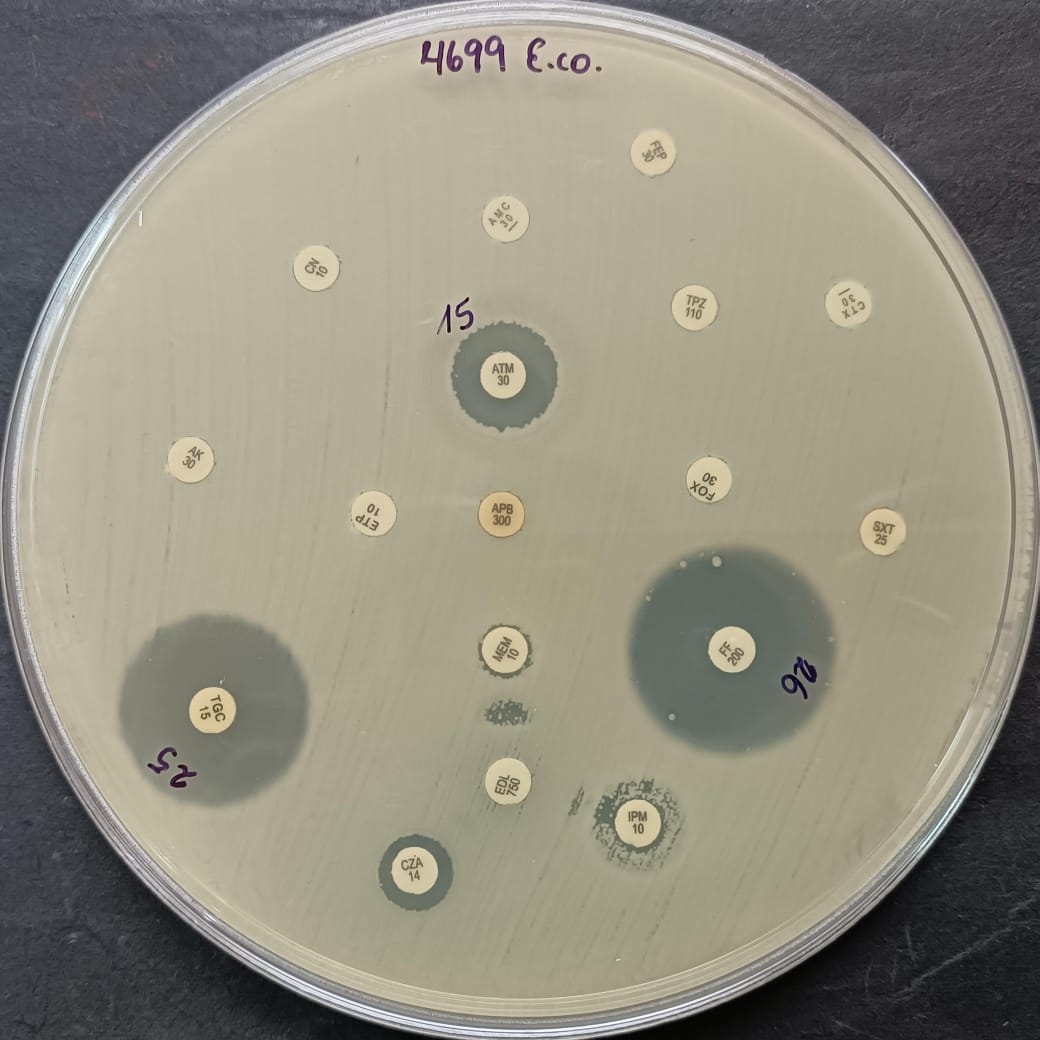


**Fuente**: Instituto Nacional de Salud del Perú

**Materia suplementaria 3**: PCR para detección de muestra aislada de carbapenemasa, en carril 7 doble producción de bla NDM, bla OXA 48 like.


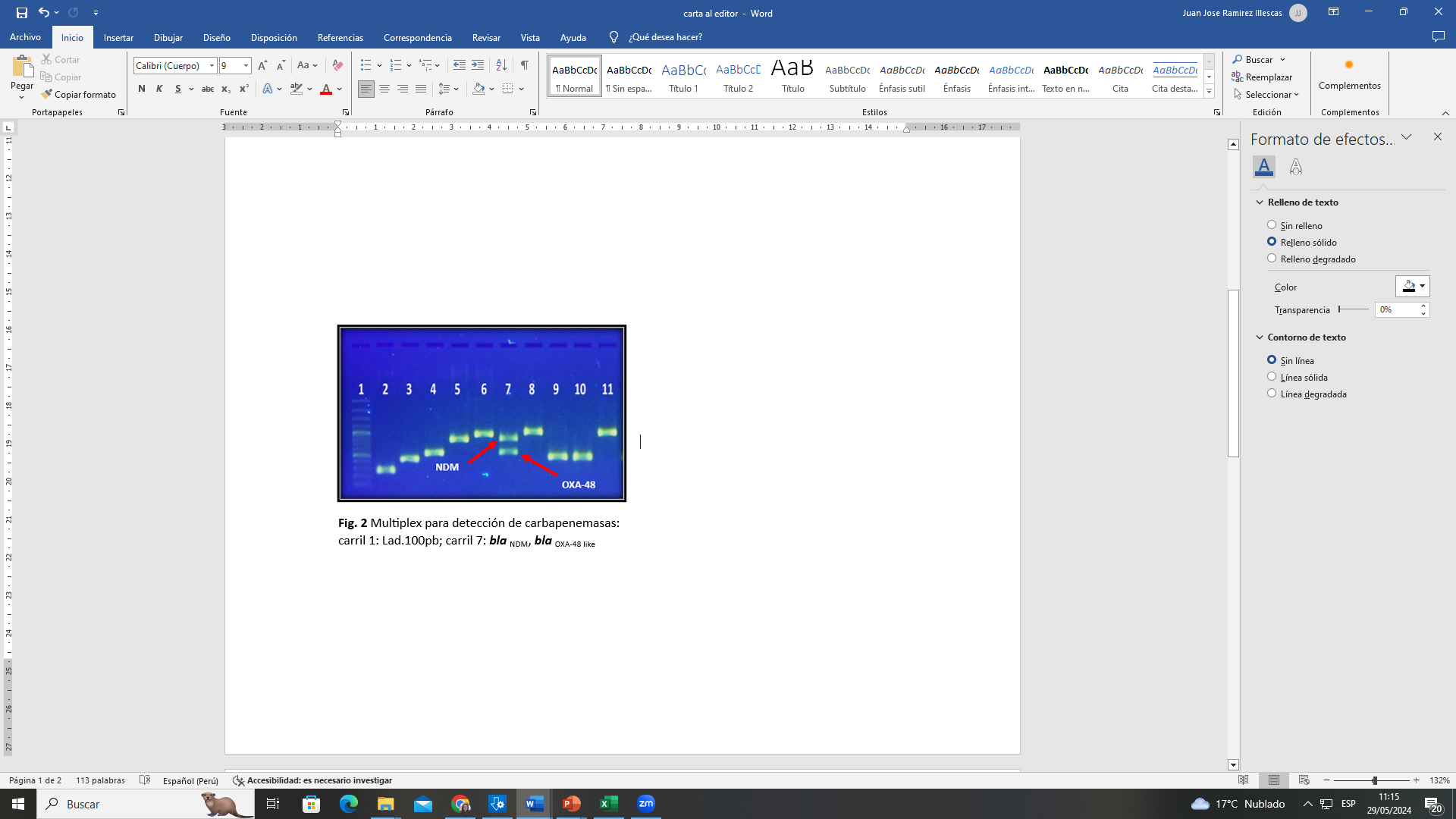


**Fuente**: Instituto Nacional de Salud del Perú
