# Supplementary material for: Isolation of Escherichia coli with a combination of carbapenemases (bla NDM +bla OXA-48-like ) in a hospital in Huancavelica, Peru
Source: Rev Peru Med Exp Salud Publica. 2025 Aug 25;42(3):333–5. doi: 10.17843/rpmesp.2025.423.14193 (PMC12679978; doi:10.17843/rpmesp.2025.423.14193)
Supplement: Translation

# Translation: Aislamiento de *Escherichia coli* con combinación de carbapenemasas (*bla*_NDM_ +*bla*_OXA-48- like_ ) en un hospital de Huancavelica, Perú

**Authors:** Dany Victorio-López, Wendi Pompilio, Jhonatan Carrera-Donayre, Juan Ramírez-Illescas, Raquel Maraza-Huarachi, Maritza Mayta-Barrios, Diana Flores-León, Martin Yagui, Luis Pampa-Espinoza

**Affiliations:** 1 Hospital Departamental de Huancavelica, Ministry of Health, Huancavelica, Perú.; 2 Clinical Bacteriology Laboratory, National Institute of Health, Lima, Perú.; 3 Strategic Interventions Unit, National Institute of Health, Lima, Perú.

Sr. Editor. Las infecciones causadas por bacterias multirresistentes constituyen un problema de salud pública a nivel mundial. En 2019, antes del inicio de la pandemia por COVID-19, se describieron 4,5 millones de muertes atribuibles a infecciones

resistentes ^(^^1^. El uso indiscriminado de antibióticos, sumado a las insuficientes medidas de control para su prescripción, ha creado el escenario perfecto para el aumento progresivo de infecciones multirresistentes en el Perú ^2^. La pandemia de COVID-19 exacerbó esta problemática, observándose que más del 50 % de los pacientes hospitalizados recibieron antibióticos sin una prescripción clara ^(^^3^. Frente a esta situación, resulta imperativo fortalecer estrategias como los programas de optimización del uso de antimicrobianos (PROA), cuyo funcionamiento ha sido reportado en solo el 28,8% de los establecimientos de salud del Perú hasta el 2022 ^4^. Del mismo modo, fortalecer las capacidades diagnósticas a través de la implementación de laboratorios con infraestructura adecuada, tecnología avanzada y pruebas microbiológicas especializadas, que permitan un diagnóstico rápido y preciso de gérmenes multirresistentes en los hospitales del Perú. Un diagnóstico oportuno, junto con un tratamiento adecuado, incrementan significativamente las probabilidades de supervivencia en pacientes adultos con infecciones complicadas ^5^, en ese contexto, presentamos el siguiente hallazgo como ejemplo del análisis microbiológico realizados en un paciente hospitalizado en un establecimiento público de salud de Perú.

El hallazgo pertenece a un paciente varón de 53 años, referido de un establecimiento de salud del distrito de Yauli en Huancavelica, debido a un cuadro de descompensación respiratoria, por lo que ingresa a la unidad de cuidados intensivos (UCI) del hospital para evaluación, manejo y monitoreo especializado. Durante su estancia se obtuvo una muestra de secreción bronquial para cultivo microbiológico. A los tres días se aíslo *Escherichia coli* con un perfil de multirresistencia a penicilinas, cefalosporinas, aminoglucósidos, fluoroquinolonas y carbapenémicos. La susceptibilidad antimicrobiana fue determinada por equipo automatizado (VITEK®). Dada la relevancia del hallazgo el equipo de laboratorio del hospital derivo la cepa bacteriana para estudio y confirmación al Laboratorio de Bacteriología Clínica del Instituto Nacional de Salud (INS) de Perú, donde se realizó la confirmación de la cepa por el sistema MALDI-TOF y los mecanismos de resistencia mediante pruebas de sinergia por difusión con discos. Finalmente se detectó la combinación de los genes de resistencia *bla*_NDM_ + *bla*_OXA-48-like_ a través del sistema multiplex PCR convencional. 

Las especies resistentes a carbapenémicos son consideradas gérmenes de prioridad critica según la lista de patógenos priorizados por la Organización Mundial de la Salud ^1^. De continuar la tendencia ascendente en las tasas de resistencia entre bacilos gramnegativos esta situación podría derivar en un escenario endémico, con tasas de mortalidad que alcanzarían hasta el 50% ^6^. Según Mayta-Barrios *et al.*, en el Perú se ha documentado un aumento de los reportes de gérmenes con resistencia a carbapenémicos en un periodo previo a la pandemia de COVID-19; además, se evidencia una creciente diseminación y expansión geográfica de estos casos. El factor de riesgo reportado fue el uso de antibióticos previo al ingreso hospitalario ^6^. En cuanto al análisis microbiológico, se siguieron las recomendaciones de la alerta epidemiológica AE-001-2022 del Centro Nacional de Epidemiología, Prevención y Control de Enfermedades ^7^. Primero, se realizaron estudios a nivel hospitalario mediante el tamizaje para la detección de carbapenemasas. Esto se llevó a cabo a partir del informe del cultivo de secreción bronquial, en el cual se identificó la presencia de una cepa de *E. coli* resistente a carbapenémicos en el antibiograma. Segundo, se realizó una prueba de sinergia con discos, mediante la cual se detectó una carbapenemasa de tipo metalo-β-lactamasa. Tercero, se confirmó la presencia de genes de resistencia mediante la reacción en cadena de la polimerasa (PCR), determinándose la existencia de genes del tipo *bla*_NDM_+ *bla*_OXA-48-like_ según análisis realizado en el INS (Tabla 1).

**Tabla 1:** Acciones propuestas y realizadas en laboratorios, hospitales, LARESA, DIRESA/DIRIS e Instituto Nacional de Salud (INS) para estudios de bacterias de prioridad critica.

Actividades	Laboratorio del hospital	Laboratorio Regional de Salud Pública y DIRESA/DIRIS	Laboratorio de Bacteriología Clínica del INS
Acciones propuestas para cada jurisdicción	Realizar estudio fenotípico manual y/o automatizado (cultivo y antibiograma). De existir resistencia a carbapenémicos (CLSI), coordinar envió de la cepa a LARESA. Implementar métodos rápidos como Blue-Carba, Carba NP directo, sinergia con discos (EDTA/APB/CZA), test de inmunolateralidad, etc.	Garantizar transporte rápido y buena conservación de envió al INS (control de calidad). Gestionar y acompañar al hospital en la gestión del presupuesto anual frente al gobierno regional para insumos de laboratorio de gérmenes resistentes. Coordinar con la unidad de epidemiologia del EESS y de DIRESA/DIRIS el reporte del brote regional de IAAS para generar alerta epidemiológica hospitalaria. Asistencia técnica temprana frente a brotes de IAAS en hospitales de su jurisdicción.	Realizar estudios confirmatorios moleculares (PCR) y confirmación de especie (MALDI-TOF) de cepas enviadas. Capacitar a equipos de microbiología de hospitales y región en métodos rápidos. Reporte y coordinación de resultados con región y hospital a través de NetLab.
Acciones realizadas en el hallazgo	Aislamiento de cultivo automatizado de *E. coli* panrresistente a antibióticos testeados como penicilinas, cefalosporinas, y carbapenems, etc. (material suplementario 1).	Recepción de la muestra del hospital y transporte al INS de Perú.	Prueba de susceptibilidad a *E. coli* y sinergia con discos (material suplementario 2) Prueba molecular PCR confirmatoria para detección de *E. coli* carbapenemasas *bla*_NDM_ + *bla*_OXA-48-like_ (material suplementario 3).

En la tabla se describen las acciones en el hallazgo de la cepa de *Escherichia coli* con resistencias a carbapenemes y doble producción de carbapenemasas. Fuente: CDC Perú ^7^.

LARESA: Laboratorio Regional de Salud Pública, DIRESA: Dirección Regional de Salud Pública, DIRIS: Dirección de Redes Integradas de Salud Lima, GORE: Gobierno Regional, EESS: establecimiento de Salud, INS: Instituto Nacional de Salud, PCR: reacción en cadena de polimerasa, IAAS: infecciones asociadas a la atención de la salud.

La combinación de carbapenemasas ha comenzado a reportarse en Latinoamérica tras la pandemia de COVID-19, lo que ha motivado la emisión de alertas epidemiológicas en la región. Esta situación, debido a su alto riesgo epidemiológico de generar brotes intrahospitalarios y asociarse con elevadas tasas de mortalidad en pacientes infectados, exige reforzar los esfuerzos diagnósticos a nivel hospitalario, con confirmación a nivel regional o nacional ^8^. En el Perú han comenzado a reportarse hallazgos de combinaciones de carbapenemasas ^9^. Esta combinación enzimática representa un riesgo adicional debido a la presencia de plásmidos en su estructura genómica, los cuales facilitan la transferencia horizontal de genes de resistencia entre distintas especies bacterianas. Por esta razón, deben reforzarse las medidas de prevención y control de infecciones, incluyendo la higiene de manos del personal de salud, las precauciones de contacto y las precauciones estándar, así como la optimización de la limpieza y desinfección de los ambientes hospitalarios. 

Para el análisis de esta cepa existió comunicación entre el laboratorio del hospital y el Laboratorio de Bacteriología Clínica del INS con la retroalimentación de los análisis confirmatorios. Es fundamental que los hospitales que cuentan con presupuesto propio y apoyo del Gobierno Regional (GORE) dispongan de un laboratorio de microbiología implementado con métodos manuales, automatizados y pruebas rápidas, como Blue-Carba, Carba NP directo, sinergia con discos (EDTA/APB/CZA), test de inmunocromatografía lateral, entre otras tecnologías de diagnóstico rápido. La disponibilidad de estas pruebas permitiría una toma de decisiones más rápida frente al paciente crítico hospitalizado, lo que podría reducir la estancia hospitalaria y mejorar la supervivencia. Para el presente reporte, se identificaron limitaciones en la información clínica debido a la naturaleza retrospectiva del análisis y a las demoras en la referencia de las muestras.

En conclusión, se requiere un compromiso real, sostenido y articulado por parte de los hospitales, las autoridades regionales y el nivel nacional, que implique un trabajo constante desde los laboratorios ante la sospecha de gérmenes de prioridad crítica. Se recomienda que los equipos de microbiología gestionen el envío oportuno de cepas prioritarias al laboratorio de referencia para su caracterización genotípica. Este esfuerzo permitirá una mejor comprensión del panorama actual de las infecciones hospitalarias causadas por bacterias multirresistentes en el Perú, y facilitará una toma de decisiones clínicas y de salud pública más oportuna, basada en la evidencia.
